# A new stoichiometric miniaturization strategy for screening of industrial microbial strains: application to cellulase hyper-producing *Trichoderma reesei* strains

**DOI:** 10.1186/1475-2859-11-70

**Published:** 2012-05-30

**Authors:** Etienne Jourdier, Laurent Poughon, Christian Larroche, Frédéric Monot, Fadhel Ben Chaabane

**Affiliations:** 1 IFP Energies nouvelles, 1 et 4 avenue de Bois-Préau, 92852, Rueil-Malmaison, France; 2 Clermont Université, Université Blaise Pascal, Institut Pascal, Polytech Clermont-Ferrand, 24 av. des Landais, BP 20206, 63174, Aubière cedex, France

**Keywords:** Screening, Miniaturization, Stoichiometry, Fed-batch, pH Control, Cellulase, *Trichoderma reesei*

## Abstract

**Background:**

During bioprocess development, secondary screening is a key step at the boundary between laboratory and industrial conditions. To ensure an effective high-throughput screening, miniaturized laboratory conditions must mimic industrial conditions, especially for oxygen transfer, feeding capacity and pH stabilization.

**Results:**

A feeding strategy has been applied to develop a simple screening procedure, in which a stoichiometric study is combined with a standard miniaturization procedure. Actually, the knowledge of all nutriments and base or acid requirements leads to a great simplification of pH stabilization issue of miniaturized fed-batch cultures. Applied to cellulase production by *Trichoderma reesei*, this strategy resulted in a stoichiometric mixed feed of carbon and nitrogen sources. While keeping the pH between shake flask and stirred bioreactor comparable, the developed shake flask protocol reproduced the strain behaviour under stirred bioreactor conditions. Compared to a an already existing miniaturized shake flasks protocol, the cellulase concentration was increased 5-fold, reaching about 10 g L^-1^. Applied to the secondary screening of several clones, the newly developed protocol succeeded in selecting a clone with a high industrial potential.

**Conclusions:**

The understanding of a bioprocess stoichiometry contributed to define a simpler and more effective miniaturization. The suggested strategy can potentially be applied to other fed-batch processes, for the screening of either strain collections or experimental conditions.

## Background

### Bioprocess development and miniaturization

During the development of an industrial process involving micro-organisms, the screening of strain collections is a compulsory step for the selection of a strain exhibiting the best performances in industrial conditions. The screened collections can be issued from any primary screening and they often contain hundreds of strains which economically cannot all be tested at a large scale. Therefore, the secondary screening step must be carried out at a small scale, but representative of industrial conditions. Any minor difference between screening method and large scale cultivation protocol may lead to the selection of a clone which is inappropriate for an industrial process [1].

**Figure 1:**
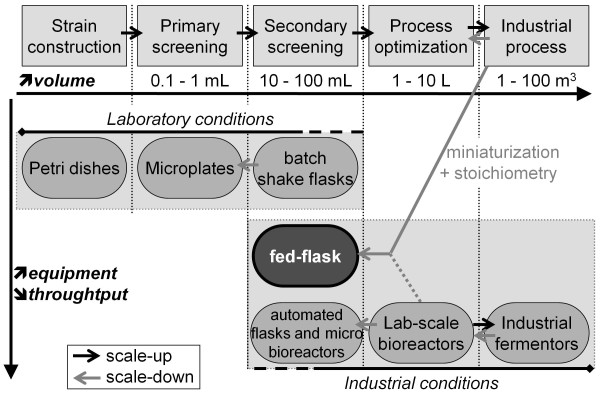
**Bioprocess development steps and available methods for each step.** Unlike batch operated shake flasks, the fed-flask protocol described here allows industrial-like operating conditions but with less equipment than microbioreactors or automated shake flasks

Current bioprocess development steps are presented in Figure 1. Strain construction is carried out on agar-based solid medium supplemented with a specific substance chosen for the screening (e.g. the substrate of the biotransformation, or a resistance marker). Then screenings are performed in liquid cultures, generally in shake flasks, but many methods have been developed to miniaturize and automate this step. Direct miniaturization of shake flasks to around 1mL can be carried out in microtiter plates (MTPs) and several high-throughput devices are commercially available for automated monitoring of growth, oxygen partial pressure and pH by optical sensors [2]. For a better control of the operating conditions (mainly pH and dissolved oxygen), microbioreactors have been developed by miniaturization of the laboratory scale bioreactor, with two distinct strategies. On the one hand, microfluidic chips have been designed to equip MTPs with micropumps for pH control and substrate feed [3]. On the other hand, stirred tank or bubble column reactors have been miniaturized down to 10 and 2 mL respectively [4,5], but these low volumes may be critical for off-line analysis.

Microbioreactors have been developed for a wide range of applications, with a material-based approach, in order to precisely mimic lab-scale bioreactors. They may be applied for high-throughput screening thanks to their low volumes but they are quite complex to handle and their operating conditions may be a limiting issue. For example, the optical sensors used for pH monitoring in microbioreactors are not useable below pH 5.5 [6], and feeding is seldom possible whereas industrial processes are frequently operated in fed-batch mode.

Recently, two simple techniques have been proposed to handle glucose-limited fed-batch in shake flask: the slow release of glucose crystals from silicon elastomer discs [7], and the enzymatic glucose release from polysaccharides [8,9]. The first one can also be used with Na_2_CO_3_ crystals for pH stabilization [10]. However, in both cases, the feed rate, although reproducible, cannot be set and controlled so that these techniques are suitable only for a primary screening. For precise fed-batch operation in shake flasks with minimum equipment, Weuster-Botz et al. proposed a parallel substrate feeding strategy in which a single pump is used to feed successively up to 16 shake flasks [11]. For pH control, a second pump can be used in the same way for base feed. However, in this case, this system requires one pH-probe and two feed lines for each flask.

Bareither and Pollard [12] recently reviewed small-scale bioreactors available on the market or in development and concluded that no single device has yet succeeded in a miniaturization which retains full functionality of conventional bioreactors. Therefore the choice or design of a miniaturized device must be based on the main characteristics of the planned industrial process.

### Cellulase production using *Trichoderma reesei*

The study case chosen for the test and validation of this strategy is cellulolytic enzymes (cellulases) production using recombinant *T. reesei* strains. *T. reesei* is a fungus with very high protein secretion capacity which has been successfully cultivated at industrial scale for cellulase production [13]. Novel industrial *T. reesei* strains are the result of genetic improvement programmes often including high-throughput screening steps [14].

Cellulase production by *T. reesei* requires an inducer of the cellulolytic system. The natural inducer cellulose, whether pure or in lignocellulosic material, leads to good productions but is hard to handle at an industrial scale because of its insolubility [15]. As a substitute, the disaccharide lactose is the most classic soluble inducer and carbon source [16]. However, even in derepressed strains, cellulase production is very low in the presence of readily-metabolized sugars in excess, which is the case in batch culture [17]. Therefore, soluble inducing carbon sources must be fed continuously, in a fed-batch or continuous protocol, so that their residual concentrations in the bioreactor will remain close to zero.

The industrial production protocol developed by IFP Energies nouvelles is based on these observations [15]. First, cellular growth is carried out in a batch mode on soluble sugar. Then, when residual sugars concentration comes to zero, cellulase production is carried out in carbon-limited fed-batch mode with concentrated lactose solution, resulting in maximized cellulase production rate. The specific substrate feed rate (equal to the specific substrate uptake rate) is the main criterion for maximized production, and it must be kept constant during scale-up or scale-down approaches. In the industrial stirred bioreactor process, temperature and dissolved oxygen are controlled, and an ammonia solution is used for pH control at 4.8 and for nitrogen supply. This protocol is too complex to be used for high-throughput screenings.

pH stabilization is a major criterion for the development of *T. reesei* small-scale cultivation protocols. It should preferably be kept between 4.0 et 5.5, like in industrial protocol. pH outside this range will stop metabolism and may durably denature produced cellulases, whose optimal pH is around 5.0. In batch shake flask cultivations without buffer, pH quickly drops below 3.5 so that many buffer systems have been tested [18,19]. The conclusion varies between authors since the chosen criterion (final enzymatic activity) depends on many factors in batch cultivations.

For the selection of a strain with high industrial potential, one of the most important criterion is the expected protein productivity so that the secondary screening must not be based solely on cellular growth. Since around 90% of the enzymatic cocktail secreted by *T. reesei* strains in industrial conditions are cellulases [20], the enzymatic activity of a supernatant is proportional to the protein concentration, and protein productivity is closely related to cellulolytic activity productivity. Thus, to free from producing cell biomass, the chosen criterion for the selection of an industrial strain is the specific protein production rate q_P_ (mg of protein per g of biomass per hour). For hyper-producing *T. reesei* strains, its value is below 5 mg_P_ g_X_^-1^ h^-1^ in batch cultivation whereas it reaches around 15 mg_P_ g_X_^-1^ h^-1^ in carbon-limited fed-batch or continuous cultivations [17]. Obviously a small-scale cultivation protocol suited for the selection of industrial strains has to allow high specific protein production rate, which requires fed-batch mode.

Recently, Cianchetta et al. described a screening strategy consistent with cultivations in batch shake flasks for the identification of hypercellulolytic *T. reesei* strains [21]. However in their shake flask protocol, the strains were cultivated with excess cellulose so the results cannot be extrapolated to the industrial carbon-limited protocol. To increase enzyme production in shake flasks, a resting cell strategy is often used, in which a lactose pulse is performed after glucose depletion, but lactose is still in excess [22]. This protocol is thereafter named shake flask pulse protocol. To our knowledge, no small-scale fed-batch protocol has been proposed for the selection of *T. reesei* strains with high industrial potential.

In this study, a stoichiometric miniaturization strategy is described for the design of a simple screening procedure which retains the experimental conditions of an industrial process. This strategy was applied to cellulase production by *T. reesei* which requires carbon-limited fed-batch and pH stabilization. For fed-batch mode, a standard miniaturization strategy was used. In parallel, a stoichiometric study of the micro-organism behaviour in industrial conditions was performed in order to develop a simple pH stabilization strategy. Usually stoichiometry is used to calculate yield or mass balance. In this work it was used for calculation of nutriments requirement for later medium design. Actually, acid or base requirements for pH control are stoichiometrically related to nutriments uptake from the culture medium. Accordingly, knowing nutriments needs allows predicting acid or base needs. In practice, no pH probe is required and base or acid are mixed with the feed, which halves the number of pumps. The resulting protocol (thereafter named "fed-flask" mode for stoichiometric fed-batch mode operated in shake flask) was first validated by comparison with reference production in bioreactor and then applied for the secondary screening of various *T. reesei* clones.

## Results

### Reference fed-batch production in bioreactor

**Figure 2:**
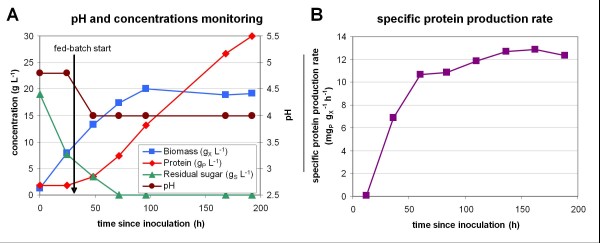
**Reference production in bioreactor.** Reference strain *T. reesei* CL847 iβ was cultivated in 750 mL bioractor following industrial protocol: batch growth phase on 20 g L^-1^ glucose for 30 h then fed-batch with 250 g L^-1^ lactose solution at 2 mL h^-1^. (**A**) pH and concentrations monitoring: cell biomass concentration (blue squares), protein concentration (red diamonds), residual sugar concentration (green triangles) and pH (brown circles). (**B**) Specific protein production rate (purple squares)

The industrial protocol already mentioned [15] was used to perform a reference fed-batch cultivation of *T. reesei* CL847 iβ strain in lab-scale bioreactor. Monitoring of pH, substrate and products concentrations (Figure 2 A), and calculation of the specific protein production rate q_P_ (Figure 2 B) showed 3 different phases. During the first 30 hours, cell growth occurred in a batch phase on glucose as a carbon source. Due to the repressor effect of glucose, no protein production was observed during this phase (q_P_ = 0 mg_P_ g_X_^-1^ h^-1^). Then, from 30 to 72 hours, the lactose feed induced protein production, but the residual sugar concentration was not null, resulting in a limited induction of the production (q_P_ = 7 mg_P_ g_X_^-1^ h^-1^). Both biomass and protein were produced. Then, after 72 hours, residual sugars concentration was null, resulting in a high induction of the protein synthesis (q_P_ = 12.5 ± 0.5 mg_P_ g_X_^-1^ h^-1^). Biomass growth stopped and protein production was maximum. During this phase, a protein production yield of 0.36 ± 0.03 g g^-1^ on lactose was obtained. Final protein concentration after 192 h total cultivation was 30 g L^-1^.

### Stoichiometry of cellulase production

To understand the nutriments needs during protein production by *T. reesei*, compositional analysis of different secretomes was performed. Measurements were done on 3 desalted secretomes produced by CL847 iβ strain in 3 independent bioreactor cultivations with lactose feed (same conditions than reference fed-batch production, duration 265, 340 and 240 h, final concentrations 43, 65 and 33 g L^-1^). These measurements were compared to the theoretical protein composition based on nucleotidic sequences of the genes coding for the four major cellulases CBH1, CBH2, EG1, EG2 [23], which account for around 75% of the secretome [20]. Results showed large differences between theoretical and measured compositions (Table 1). Whereas carbon and hydrogen content were quite equivalent, oxygen content was 38% higher and nitrogen and sulfur content were 24% and 38% lower, respectively, in the measured composition compared to the theoretical one. Using mean measured secretome composition and measured production yield, the overall stoichiometry of protein production by *T. reesei* on lactose when no residual sugars (no biomass growth) was determined as:

(1) $${\text{CH}}_{1.833}O_{0.917}+0.597O_{2}+0.093{\text{NH}}_{3}↵0.400{\text{CH}}_{1.733}O_{0.508}N_{0.23}+0.600{\text{CO}}_{2}+0.708H_{2}O$$

**Table 1:** **Theoretical and measured compositions of*T. reesei*secretome**

	**theoretical**	**measured**	
	**mass content**	**mass content**	**molar composition**
**C**	53.0 ± 0.9%	47.3 ± 0.1%	1
**H**	6.6 ± 0.1%	6.83 ± 0.04%	1.733
**O**	23.2 ± 0.7%	32.0 ± 0.8%	0.508
**N**	16.7 ± 0.2%	12.7 ± 0.5%	0.23
**S**	1.6 ± 0.5%	1.00 ± 0.05%	0.008
**Total**	100.1 ± 2.4%	99.7 ± 0.6%	

Three independent final secretomes produced by *T. reesei* CL847 iβ under reference fed-batch production in bioreactor (with lactose feed) were desalted and analysed for their elementary compositions (values are mean and standard deviation of the 3 secretomes). Theoretical composition was based on amino-acids sequences of the fourth main cellulases.

During protein production on lactose, no other metabolite such as an organic acid is consumed or produced, so this equation describes all the biological reactions occurring during protein production. For instance, using this equation, the calculated carbon balance for the third phase of the reference bioreactor production (After 72 h: protein production with no growth) was 0.95 gC_produced_ gC_consumed_^-1^, which proves that every carbon substrate and product were considered.

### Fed-flask protocol design

For the design of the new fed-flask protocol, a standard miniaturization of the lab-scale bioreactor was first carried out at constant specific substrate feed rate (Table 2). Flasks were chosen for their simplicity to handle and rotary shaker for easy agitation and temperature control. After preliminary experiments, working volume (50 mL) and biomass concentration (7 to 8 g L^-1^) were chosen to avoid oxygen transfer limitation (see Discussion). Initial glucose concentration for batch phase (15 g L^-1^) was chosen to reach the biomass concentration. Temperature was not a critical issue since the optimal range for *T. reesei* is wide [15]. Feed rate and lactose concentration in feed were chosen to keep constant the specific substrate feed rate.

**Table 2:** Conditions used for the stoichiometric miniaturization strategy

			**bioreactor protocol**	**fed-flask protocol**	**miniaturization fold**
Working volume (mL)			750	50	/15
Biomass concentration (g L^-1^)			17	7.5	/2.2
Feed composition	lactose (g L^-1^)		250	50	/5
	NH_3_ (mM)		(separate^1^)	160	
Feed rate (mL h^-1^)			2	0.3	/6.6
specific lactose feed rate (g g^-1^ h^-1^)			0.04	0.04	constant

**Figure 3:**
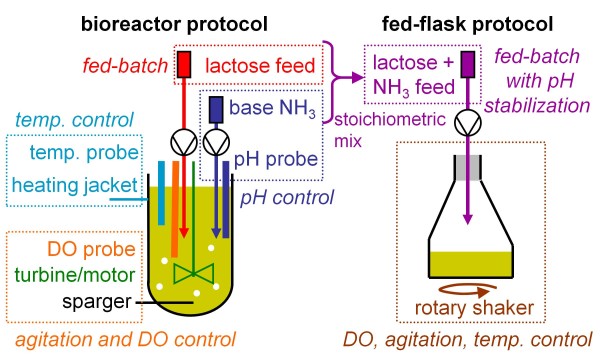
**Stoichiometric miniaturization strategy: principle diagram.** To reduce needed equipment, substrate feed and base ammonia were stoichiometrically mixed in a single solution. This allows pH stabilization with halved pump number and no pH probe

Experimental conditions for reference fed-batch production in bioreactor, and miniaturization fold applied for the design of the fed-flask protocol (at constant specific lactose feed rate). ^1^For bioreactor protocol, NH_3_ was added on demand by pH control system, whereas it was stoichiometrically mixed with the feed for fed-flask protocol (see Figure 3).

Then, a stoichiometric approach was used to remove pH control system by mixing the base with the substrate feed (Table 2 and Figure 3). With this strategy, no pH probe was required and the number of pumps was halved. The measured stoichiometry of protein production on lactose was used to predict the NH_3_ base need during fed-batch: 3.2 mmol of NH_3_ per gram of lactose. Using 50 g L^-1^ lactose feed, 15 mL of 11 N ammonia solution were mixed per liter of lactose feed. With the same reasoning, sulfur requirement was calculated to be 0.8 g L^-1^ ammonium sulfate.

### Validation of the fed-flask protocol

**Figure 4:**
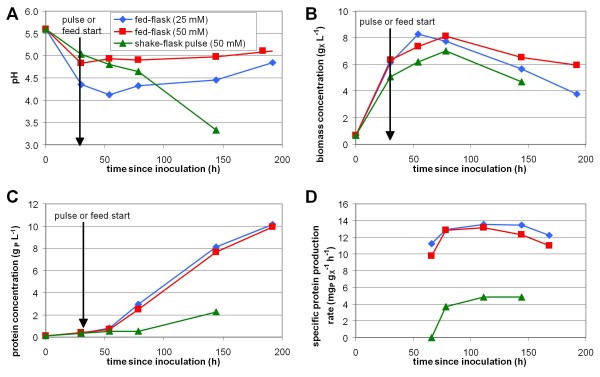
**Validation of the miniaturized fed-flask protocol design.** Reference strain *T. reesei* CL847 iβ was cultivated in 50 mL shake-flask following shake flask pulse protocol (green triangles), or fed-flask protocol buffered with 25 (blue diamonds) or 50mM (red squares). pH (**A**), biomass concentration (**B**) and protein concentration (**C**) monitoring, and specific protein production rate calculation (moving average) (**D**)

Using the reference strain *T. reesei* CL847 iβ, the new fed-flask protocol was compared to the shake flask pulse protocol and to the reference fed-batch production protocol in bioreactor (previously presented Figure 2). For flasks experiments, growth phase was identical, then production was induced with a pulse of 20 g L^-1^ lactose (shake flask pulse protocol) or with a stoichiometric mixed feed of lactose and ammonia (new fed-flask protocol). Off-line monitoring of pH and concentrations, and calculation of specific protein production rate are presented in Figure 4.

During growth phase on glucose, pH felt because of ammonium consumption (Figure 4 A). This fall was weaker when the culture medium contained 50 mM phtalate than 25 mM owing to the buffer effect of phtalate. However, in shake flask pulse protocol, even 50 mM phtalate buffer was insufficient to stabilize pH after the lactose pulse. pH dropped below 3.3 after 142 h and biological activity stopped due to low pH effect. With the new fed-flask protocol, pH remained constant at 5.0 in 50 mM buffered flask and slightly rose from 4.1 to 4.8 in 25 mM buffered flasks. The use of a feed mixing lactose and ammonia in stoichiometric proportion mimicked the pH control strategy used for reference fed-batch production in bioreactor and allowed a good pH stabilization in flasks for more than 150 h.

Biomass and protein concentration profiles were similar in the new fed-flask protocol and in the reference fed-batch bioreactor cultivation (Figure 4 B and C, compared to Figure 2 A). After feed start, biomass remained constant (in mass) and protein production was almost linear, reaching 10 g L^-1^ after 160 h of feeding. No effect of buffer concentration or pH was observed on the protein production rate. With the shake flask pulse protocol with lactose pulse, protein production rate was 3.5 times lower, and stopped 110 h after the pulse at 2.3 g L^-1^ due to pH fall. Final lactose concentration was 14.8 g L^-1^, only 5.2 g L^-1^ lactose had been consumed before low pH inhibition occurred.

**Figure 5:**
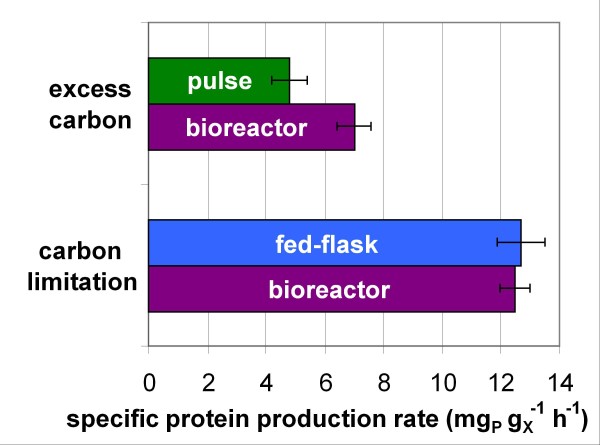
**Comparison of*T. reesei*CL847 iβ reference strain behaviour under three different cultivation protocols.** Mean specific protein production rate (during production phase) was measured in reference bioreactor protocol (purple), shake flask pulse protocol (green), and new fed-flask protocol (blue)

The specific protein production rate (Figure 4 D) was limited to 4.8 mg_P_ g_X_^-1^ h^-1^ in the shake flask pulse protocol due to the excess of lactose. In contrast, in the new fed-flask protocol, it reached 12.7 ± 0.8 mg_P_ g_X_^-1^ h^-1^, owing to the carbon-limited feed which maintained residual sugar concentration close to zero (no residual sugar was detected by HPLC). Both values were consistent with those obtained in reference fed-batch bioreactor cultivation performed under comparable substrate feeding conditions (Figure 5). These results validated the new fed-flask protocol for the screening of strains with high hyperproducing capacity.

### Application to secondary screening

To test this new protocol, a strain collection issued from a genetic engineering work was screened in order to rank the strains according to their protein production capacity in the operational conditions defined during the miniaturization.

**Figure 6:**
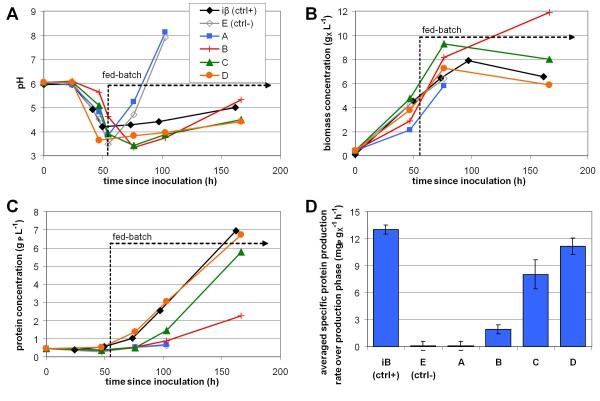
**Application of the miniaturized fed-flask protocol to clone screening.** Reference strain *T. reesei* CL847 iβ (black diamonds), and five recombinant strains: negative control E (gray diamonds) and clones A,B,C,D (respectively in blue squares, red cross, green triangles, orange circles), were cultivated in 50 mL shake flasks following fed-flask protocol: growth phase on 15 g L^-1^ glucose for 50 h then fed-batch with mixed lactose and ammonia feed (see Table 2). pH (**A**), biomass (**B**) and protein (**C**) monitoring, and specific protein production rate (**D**). Specific protein production rates were calculated over production phase (at constant biomass) and are mean and standard deviation of two independent replicates

During conidia purification and isolation steps, 25 clones were selected for stability, growth and general morphology on PDA plates. Then, a primary screening was achieved in classic shake flasks to check for transgene expression and protein production. For the secondary screening with the new fed-flask protocol, 4 clones (A, B, C, D) were selected for their good transgene expression and fair protein production levels (data not shown). These 4 clones were considered equivalent at the end of the primary screening. Clone E which lost the parental strain capacity to produce high levels of proteins was selected as a negative control. Parental strain *T. reesei* CL847 iβ was selected as a positive control. These 6 clones were tested by using the new fed-flask protocol: feed was started after 50 h growth phase on 15 g L^-1^ glucose. The experiments were duplicated and gave similar results: Figure 6 presents the monitoring of the second experiment and average specific protein production rate.

During growth phase, the 6 clones showed differences in pH decrease (Figure 6 A). Since the buffer concentration was identical, these differences may reflect different growth rates, or different spore concentration at inoculation. During the fed-batch phase, clones B, C and D showed a pH profile similar to the reference strain *T. reesei* CL847 iβ, with a good pH stabilization, between 4 and 5. On the other hand, clone A and negative control E showed a very quick rise of pH, up to 8, which completely inhibited biomass growth and protein production.

Biomass and protein profiles (Figure 6 B and C) were similar for clones C and D and for reference strain *T. reesei* CL847 iβ, with almost constant biomass concentration around 7 g L^-1^, and linear protein production up to around 7 g L^-1^ after 120 h feed. Clone B showed biomass accumulation up to 12 g L^-1^ and lower protein production, reaching 2.3 g L^-1^. Clone A and negative control E did not produce any protein.

The mean specific protein production rate (Figure 6 D) is the only reliable criterion to determine whether a strain is a hyperproducing one or not. Clones A and B, like negative control E, lost the hyperproducing phenotype of the parental reference strain iβ. Clone D has almost the same specific protein production rate as the parental strain: it kept the hyperproducing phenotype. Clone C, which produced almost the same protein concentration, but with higher producing biomass concentration, has an intermediate phenotype, with a specific protein production rate 38% lower.

Using this new fed-flask protocol for a secondary screening step, 5 clones were ranked according to their protein production capacity, which was not possible during the first screening step with classic shake flasks protocol.

## Discussion

### Compositional analysis of *T. reesei* secretomes

Using three different secretomes produced by *T. reesei* in bioreactor fed-batch cultivations, the mean nitrogen mass content was measured at 12.7 ± 0.5% instead of the usual 16%. This result can be explained by the high glycosylation level of *T. reesei* cellulases [24]. For example, Hui et al. studied CBH2, EG1 and EG2 glycosylation patterns and determined that glycosylation accounted for 12 to 24% of their molecular mass [25]. Here, using mean theoretical cellulase composition and mean glycosylation composition, glycosylation was estimated to account for around 25% of the molecular mass of produced cellulases, which is consistent with Hui et al.

### Protocol design

In order to design a small-scale protocol for secondary screening, the industrial fed-batch protocol had to be simplified as much as possible. In particular the complex pH control system, with pH probe and concentrated ammonia solution, had to be removed. Since the use of a lactose limited feed was compulsory to maintain maximal protein production, a new miniaturization strategy was proposed, based on the stoichiometric mix of carbon substrate and ammonia in the feed. Protein production stoichiometry in fed-batch cultivation was used to calculate the ammonia needed which was introduced with the lactose feed. This strategy was successful for pH stabilization and this result confirms that the stoichiometric equation used well describes protein production, especially because no organic acid or other ionic metabolite is produced. However, since this stoichiometry may slightly vary according to culture conditions, and since the growth phase is carried out without feed so without ammonia input, the culture medium needed to be buffered.

During fed-flask protocol validation, similar protein productions were observed in media buffered with 25 mM or 50 mM phtalate. Although buffering is crucial in flasks experiments, a weaker buffer system was chosen for the screening experiments, with 25 mM phtalate, since high buffer concentration may have an effect on cells behaviour [18]. Moreover, pH drop during growth phase is a good indicator of sugar consumption, which is the major criterion for deciding to start the feeding. During production phase, pH is mainly stabilized by the stoichiometric ammonia input from feed, so buffer concentration is less important. However, a pH drop below 3.5, as observed in shake flask pulse protocol, may inactivate produced enzymes.

### Oxygen transfer

Due to their low K_L_a values, oxygen transfer is often considered as the main issue in shake flasks, especially for high cell density experiments [12]. In preliminary experiments, slower growth was observed in 100 mL working volume compared to 50 mL, due to oxygen transfer rate limitation (data not shown). Therefore a 50 mL working volume was chosen. During the fed-batch step, the volume increased but the oxygen demand was lower than in the growth phase since the carbon source supply was limited by the feed rate. So, oxygen was not a limiting nutriment during fed-batch, which is consistent with the fact that lactose residual concentration remained close to zero.

To validate this hypothesis, we calculated the minimal K_L_a needed to allow sufficient oxygen transfer during production phase. Using measured protein production stoichiometry and set lactose feed rate in our experimental conditions (Table 2), the oxygen consumption rate was calculated to be 200 mg_O2_ L^-1^ h^-1^. Then the minimum K_L_a value (for null dissolved oxygen concentration) was estimated at a value of 25 h^-1^. Every experimental device allowing K_L_a above this value will be sufficient for our experimental conditions. For shake flasks, this value is consistent with K_L_a measured by Wittman et al. [26]: around 80 h^-1^ in shake flasks with 50 mL water at 150 rpm.

Oxygen transfer in flasks is a critical issue when designing fed-batch mode which maintains substrate limitation. The simple calculation described above for the minimal K_L_a value needed can direct the choice of the experimental device.

### pH monitoring

Clone A and negative control E did not produce any protein and showed a quick pH rise after lactose feed start. They might have lost the ability to metabolize lactose, which accumulated in the culture medium (up to 12 g L^-1^ at 100 h, whereas residual concentration remained null for clones B to D). Then nitrogen from feed was not co-metabolized and ammonia also accumulated in the medium (up to 85 mM at 100 h, whereas it remained constant at around 40 mM for clones B to D), leading to this quick pH rise. Clone B used lactose feed for growth instead of protein production, with similar mass yield on lactose. But the amount of nitrogen needed for growth is lower than the one used for protein production (Harima et Humphrey measured a nitrogen mass content of 6.7% for *T. reesei* QM9414 strain [27]). In our miniaturization strategy, ammonia input was based on nitrogen content in proteins, so clone B accumulated ammonia in culture medium (up to 50 mM at 166 h), which explains the quicker pH rise for clone B cultivation than for positive control CL847 iβ strain. Then a pH rise quicker than positive control may be a good indicator to eliminate clones with lower enzyme producing capacity.

### Fed-flask protocol advantages

The comparison of the flasks protocols using reference strain *T. reesei* CL847 iβ (Figure 5) showed that protocols in flasks which do not handle the carbon limitation are not suitable to rank hyperproducing strains according to their specific protein production rate, whereas the fed-flask protocol did. It will be essential after genetic engineering works to quickly and reliably select strains which will have the best productivity at an industrial scale.

Moreover this new fed-flask protocol requires much less equipment than bioreactor cultivation: no bioreactor, only flasks and rotary shaker; no pH control system (neither pH probe nor base nor additional controlled pump); no dissolved oxygen control system (neither oxygen probe nor compressed air nor motor). In addition, the number of pumps for feeding can be reduced using the parallel feeding system developed by Weuster-Botz et al. [11]. Thereby it allows efficient secondary screening of collections of strains. We estimated that an operator who needed 2 weeks to carry out 4 bioreactor cultivations in parallel, can carry out 16 fed-flask cultivations per week, that is, a 8-fold increase.

### Miniaturization strategy

The miniaturization strategy proposed in this study was developed to mimic an industrial process. It may be applied for the miniaturization of many other industrial biotechnological processes. Its main advantage is to allow pH stabilization in flasks when operated in carbon-limited fed-batch mode. Therefore this strategy is suitable for every fed-batch process, provided that pH variation can be related to the consumption of the substrate present in the feed. In this study, a stoichiometric approach was used to determine the relation between lactose and ammonia consumption, but an empirical approach can also be used for this determination. In the general case, even overflow metabolites as organic acids may be considered, as long as their production is stoichiometrically linked to substrate consumption.

Moreover, this miniaturized protocol can be used for other applications. Unlike strategies based on diffusional or enzymatic release, this strategy can be applied to complex feed compositions and to accurate feed rate profiles, provided that the pump system allows precise rate control. Therefore the resulting protocol can also be used to test the effect of the feed composition or the feed rate on the behaviour of a given strain.

## Conclusion

A new strategy has been proposed for the design of screening protocols, which ensures a simpler and more accurate miniaturization of fed-batch operated bioprocess. Based on a stoichiometric study of the bioprocess, the feed composition may be designed to supply every nutriment and base or acid requirements in a single solution. Its main advantage is to enable a good pH stabilization without control system. Therefore strain behaviour is identical to pH-regulated bioreactor cultivation so that the screening is carried out in industrial-like conditions. Moreover, less equipment is needed for the screening of strain collection or experimental conditions, since it does not require any pH probe or additional pump.

This strategy was applied to cellulase production by *T. reesei*, a key step for the biochemical conversion of lignocellulosic materials. The resulting protocol was validated using reference fed-batch production in bioreactor then applied to the secondary screening of several clones in bioreactor-like operational conditions. It succeeded in selecting an engineered hyper-producing strain with high industrial potential.

## Methods

### Compositional analysis

To determine the stoichiometry of protein production by *T. reesei*, compositional analysis of several supernatants was realized by SGS Multilab (Evry, France). To avoid concurrent measurement of remaining ions from culture medium, supernatants were first desalted using FPLC (Akta; GE Healthcare) equipped with Hitrap desalting column, then lyophilized.

### Culture media

For bioreactor cultivations, the medium composition was: cornsteep solid 4 g L^-1^; KOH 1.66 g L^-1^; H_3_PO_4_ 85% 2.5 mL L^-1^; (NH_4_)_2_SO_4_ 2.8 g L^-1^; MgSO_4_,7H_2_O 0.6 g L^-1^; CaCl_2_,2H_2_O 0.6 g L^-1^; FeSO_4_-7H_2_O 60 mg L^-1^; MnSO_4_,H_2_O 12 mg L^-1^; ZnSO_4_,7H_2_O 16 mg L^-1^; CoNO_3_,6H_2_O 18 mg L^-1^; H_3_BO_3_ 2 mg L^-1^. pH is adjusted to 4.8 with NH_3_ 20%.

For shake flasks, fed-flasks, and preculture cultivations, the media composition was: cornsteep solid 1.5 g L^-1^; dipotassium phtalate 6 g L^-1^ (case 25 mM) or 12 g L^-1^ (case 50 mM); H_3_PO_4_ 85% 0.8 mL L^-1^; (NH_4_)_2_SO_4_ 4.2 g L^-1^; MgSO_4_,7H_2_O 0.3 g L^-1^; CaCl_2_,2H_2_O 0.15 g L^-1^; FeSO_4_-7H_2_O 30 mg L^-1^; MnSO_4_,H_2_O 6 mg L^-1^; ZnSO_4_,7H_2_O 8 mg L^-1^; CoNO_3_,6H_2_O 9 mg L^-1^; H_3_BO_3_ 1 mg L^-1^. pH was adjusted to 6.0 with NaOH 30%.

### Strains

*T. reesei* CL847 iβ was obtained by genetic engineering of the hyperproducer CL847 strain, both from Cayla Company, Toulouse France [28,29]. Clones used to test the new protocol were obtained by genetic engineering from *T. reesei* CL847 iβ strain. All strains were maintained on PDA plates.

### Bioreactor cultivations

For a standard fed-batch production based on the industrial protocol [30], cultivation was carried out in Dasgip fedbatch-pro bioreactors with an initial working volume of 750 mL. Growth phase in batch was performed on 20 g L^-1^ glucose at pH 4.8 and 27 °C for 30 h. Then fed-batch was performed at pH 4.0 and 25 °C with 250 g L^-1^ lactose solution feed at 2 mL h^-1^. pH was automatically adjusted with 5.5 N NH_3_ solution. Aeration rate was fixed at 30 sL h^-1^ and agitation was regulated to maintain 40% dissolved oxygen.

### Flasks cultivations

500 mL Schott flasks with 50 mL working volume were incubated at 30 °C and 150 rpm in an Infors rotary shaker.

For shake flask pulse protocol, the culture medium was buffered with 50 mM dipotassium phtalate. Growth phase was performed on 15 g L^-1^ glucose for 33 h. Then protein production was induced with a 20 g L^-1^ lactose pulse.

The new fed-flask protocol was designed by stoichiometric miniaturization of the bioreactor protocol (see text, Table 2, and Figure 3), for an initial working volume of 50 mL and a biomass concentration around 8 g L^-1^. Growth phase was performed on 15 g L^-1^ glucose for 50 h (until glucose concentration below 5 g L^-1^) then flasks were fed at 0.3 mL h^-1^ with a mixed solution containing: lactose 50 g L^-1^; NH_3_ 20% (11 N) 15 mL L^-1^; (NH_4_)_2_SO_4_ 0.8 g L^-1^. This composition was calculated from the stoichiometry of protein production to meet carbon, nitrogen and sulfur requirements, and to stabilize pH.

### Analysis

Culture medium was filtrated using Whatman GF/C filters. For biomass concentration determination, biomass cake was washed with distilled water then dried at 105 °C until constant weight . For protein concentration determination, supernatants were diluted with distilled water then protein concentration was measured against BSA standard (0-1.5 g L^-1^ range with second-order regression) by Lowry method [31] using DC Protein Assay (Biorad). Sugars concentration was measured by HPLC: separation was carried out using Varian Metacarb 87P column with mobile phase milliQ water at 0.4 mL min^-1^, 80 °C and pressure around 32 bar; detection was carried out with Waters 2414 refractive index detector. Ammonium concentration was measured by HPLC: separation was carried out using Dionex IonPac CS12A column with mobile phase methanesulfonic acid 20 mM at 1 mL min^-1^, 30°C and pressure around 2000 psi; detection was carried out with Dionex CD conductivity detector at 35°C.

## Competing interests

IFP Energies nouvelles has filed a patent application on this methodology.

## Authors' contributions

EJ and FBC designed the study. EJ carried out the experiments and drafted the manuscript. CL and LP are academic supervisers of EJ. All authors revised, read and approved the final manuscript.
